# (2*E*)-2-Hydroxy­imino-*N*′-[(*E*)-2-pyridyl­methyl­ene]propanohydrazide

**DOI:** 10.1107/S1600536809034400

**Published:** 2009-09-09

**Authors:** Yurii S. Moroz, Valentina A. Kalibabchuk, Elżbieta Gumienna-Kontecka, Viktor V. Skopenko, Svetlana V. Pavlova

**Affiliations:** a Department of Chemistry, Kyiv National Taras Shevchenko University, Volodymyrska Str. 64, 01601 Kyiv, Ukraine; b Department of General Chemistry, O.O. Bohomolets National Medical University, Shevchenko blvd. 13, 01601 Kiev, Ukraine; c Faculty of Chemistry, University of Wrocław, 14 F. Joliot-Curie str., 50-383 Wrocław, Poland

## Abstract

In the title compound, C_9_H_10_N_4_O_2_, the pyridine ring is twisted by 16.5 (1)° from the mean plane defined by the remaining non-H atoms. An intra­molecular N—H⋯N inter­action is present. In the crystal, inter­molecular O—H⋯N and N—H⋯O hydrogen bonds link mol­ecules into layers parallel to the *bc* plane. The crystal packing exhibits π–π inter­actions indicated by the short distance of 3.649 (1) Å between the centroids of the pyridine rings of neighbouring mol­ecules.

## Related literature

For the crystal structures of related oxime derivatives, see: Mokhir *et al.* (2002 ▶); Moroz *et al.* (2009 ▶). For 2-hydroxy­imino­propanamide and amide derivatives of 2-hydroxy­imino­propanoic acid, see: Onindo *et al.* (1995 ▶); Duda *et al.* (1997 ▶); Sliva *et al.* (1997*a* ▶). For the preparation and characterization of 3*d*-metal complexes with the structural analog of the title compound, see: Moroz *et al.* (2008*a* ▶,*b* ▶). For the synthesis of 2-(hydroxy­imino)propane­hydrazide, see Fritsky *et al.* (1998 ▶).
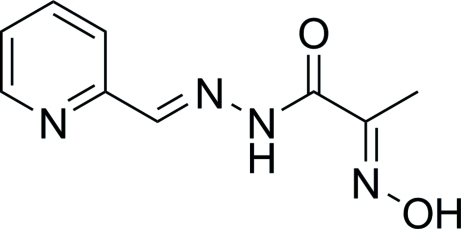


## Experimental

### 

#### Crystal data

C_9_H_10_N_4_O_2_
*M* *_r_* = 206.21
Monoclinic, 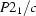

*a* = 10.274 (4) Å
*b* = 9.717 (4) Å
*c* = 10.136 (4) Å
β = 109.28 (4)°
*V* = 955.2 (7) Å^3^
*Z* = 4
Mo *K*α radiation
μ = 0.11 mm^−1^
*T* = 100 K
0.4 × 0.4 × 0.3 mm

#### Data collection

Kuma KM-4-CCD diffractometer
Absorption correction: none
10648 measured reflections
2680 independent reflections
2449 reflections with *I* > 2σ(*I*)
*R*_int_ = 0.023

#### Refinement

*R*[*F* ^2^ > 2σ(*F* ^2^)] = 0.039
*wR*(*F* ^2^) = 0.106
*S* = 1.08
2680 reflections
176 parameters
All H-atom parameters refined
Δρ_max_ = 0.46 e Å^−3^
Δρ_min_ = −0.18 e Å^−3^

### 

Data collection: *CrysAlis CCD* (Oxford Diffraction, 2006 ▶); cell refinement: *CrysAlis RED* (Oxford Diffraction, 2006 ▶); data reduction: *CrysAlis RED*; program(s) used to solve structure: *SHELXS97* (Sheldrick, 2008 ▶); program(s) used to refine structure: *SHELXL97* (Sheldrick, 2008 ▶); molecular graphics: *ORTEP-3 for Windows* (Farrugia, 1997 ▶); software used to prepare material for publication: *WinGX* (Farrugia, 1999 ▶).

## Supplementary Material

Crystal structure: contains datablocks I, global. DOI: 10.1107/S1600536809034400/cv2608sup1.cif

Structure factors: contains datablocks I. DOI: 10.1107/S1600536809034400/cv2608Isup2.hkl

Additional supplementary materials:  crystallographic information; 3D view; checkCIF report

## Figures and Tables

**Table 1:** Hydrogen-bond geometry (Å, °)

*D*—H⋯*A*	*D*—H	H⋯*A*	*D*⋯*A*	*D*—H⋯*A*
O2—H21⋯N1^i^	0.881 (17)	1.897 (18)	2.7667 (17)	168.6 (15)
N3—H31⋯O1^ii^	0.879 (16)	2.217 (16)	2.9656 (15)	142.9 (14)
N3—H31⋯N4	0.879 (16)	2.240 (16)	2.6059 (15)	104.7 (12)

Symmetry codes: (i) 
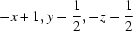
; (ii) 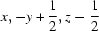.

**Fig. 1.**
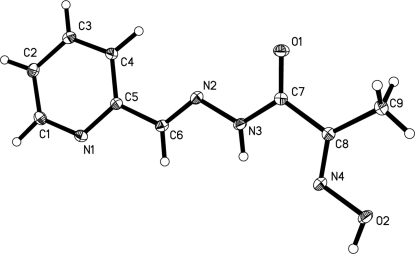
A view of 1, with displacement ellipsoids shown at the 40% probability level and atom labelling.

## supplementary crystallographic information

### Comment

As a part of our research study we present the structure of the title compound, 1 (Fig. 1), which comprises several donor groups: oxime, hydrazone, azomethine, and pyridine. It has been shown previously that structurally similiar strand ligand forms mono- and tetranuclear grid-like assemblies with 3d-metal ions (Moroz *et al.*, 2008*a*,*b*).

The C—N and N—O bond lengths in the oxime group, *i.e.* 1.285 (1) and 1.387 (1) Å, respectively, adopt typical values (Mokhir *et al.*, 2002; Moroz *et al.*, 2009). The oxime group is in a *trans* position with respect to the amide group, in accordance with the structures of 2-hydroxyiminopropanamide and other amide derivatives of 2-hydroxyiminopropanoic acid (Onindo *et al.*, 1995; Duda *et al.*, 1997; Sliva *et al.*, 1997*a*). This conformation is stabilized by an N3—H···N4 intramolecular interaction (Table 1). The CH_3_C(=NOH)C(O)NH fragment deviates from planarity because of a twist between the oxime and amide groups about the C7—C8 bond; the O1—C7—C8—N4 torsion angle is 173.3 (1)°. The C—N bond length in the azomethine group is 1.283 (1) Å, and the N2—C6—C5 angle has almost ideal value 120.5 (1)°. The pyridine nitrogen atom is situated in an *anti*- position with respect to the azomethine group.

In the crystal packing, molecules are connected by O2—H···N1 and N3—H···O1 hydrogen bonds (Table 1), where the oximic oxygen and hydrazone nitrogen atoms act as donors and the hydrazone oxygen and pyridine nitrogen atoms act as acceptors. Due to the presence of the system of O2—H···N1 and N3—H···O1 hydrogen bonds, layers parallel to *bc* plane are formed. The layers are connected in three-dimensional structure by π-π contacts with centroid-to-centroid distance of 3.649 (2) Å, which arise between the pyridine rings of neighbour molecules.

### Experimental

The compound 1 has beeen prepared accourding to following procedure: picolinaldehyde (1.1 ml, 0.012 mol) was added to 20 ml of a stirred warm ethanol/water solution of 2-(hydroxyimino)propanehydrazide (1.17 g, 0.01 mol) prepared according to early reported method (Fritsky *et al.*, 1998). After 20 minutes of stirring at 60°C a yellowish precipitate was formed. It was filtered off, washed with water and acetone and recrystalized from methanol. Yield: 1.65 g, 80%. ^1^H NMR, 400.13 MHz, (DMSO-*d_6_*): 1.983 (s, 3H, CH_3_), 7.393 (dd, 1H, py-5, J = 5.1 Hz, J = 7.2 Hz, py—H), 7.854 (t, 1H, py-4, J = 7.2 Hz, py—H), 7.932 (d, 1H, py-3, J = 7.2 Hz, py—H), 8.494 (s, 1H, CH), 8.594 (d, 1H, py-6, J = 5.1 Hz, py—H), 11.703 (s, 1H, NH), 11.932 (s, 1H, NOH); IR (KBr, cm^-1^): 1670 ν(CO_amid I_), 1040, 895 ν(NO_oxim_), 3345 ν(NH_as_). Anal. calc. for C_9_H_10_N_4_O_2_ C, 52.42; H, 4.89; N, 27.17. Found: C, 52.30; H, 4.98; N, 26.98.

### Refinement

All H atoms were located in a difference Fourier map and refined isotropically.

### Crystal data

**Table:**

C_9_H_10_N_4_O_2_	*F*(000) = 432
*M**_r_* = 206.21	*D*_x_ = 1.434 Mg m^−^^3^
Monoclinic, *P*2_1_/*c*	Mo *K*α radiation, λ = 0.7107 Å
Hall symbol: -P 2ybc	Cell parameters from 11569 reflections
*a* = 10.274 (4) Å	θ = 3–37°
*b* = 9.717 (4) Å	µ = 0.11 mm^−^^1^
*c* = 10.136 (4) Å	*T* = 100 K
β = 109.28 (4)°	Block, white
*V* = 955.2 (7) Å^3^	0.4 × 0.4 × 0.3 mm
*Z* = 4	

### Data collection

**Table:**

Kuma KM-4-CCD diffractometer	2449 reflections with *I* > 2σ(*I*)
Radiation source: Enhance (Mo) X-ray Source	*R*_int_ = 0.023
graphite	θ_max_ = 30.0°, θ_min_ = 3.2°
Detector resolution: 8.3359 pixels mm^-1^	*h* = −14→13
ω scans	*k* = −13→10
10648 measured reflections	*l* = −14→14
2680 independent reflections	

### Refinement

**Table:**

Refinement on *F*^2^	Primary atom site location: structure-invariant direct methods
Least-squares matrix: full	Secondary atom site location: difference Fourier map
*R*[*F*^2^ > 2σ(*F*^2^)] = 0.039	Hydrogen site location: inferred from neighbouring sites
*wR*(*F*^2^) = 0.106	All H-atom parameters refined
*S* = 1.08	*w* = 1/[σ^2^(*F*_o_^2^) + (0.0621*P*)^2^ + 0.2439*P*] where *P* = (*F*_o_^2^ + 2*F*_c_^2^)/3
2680 reflections	(Δ/σ)_max_ < 0.001
176 parameters	Δρ_max_ = 0.46 e Å^−^^3^
0 restraints	Δρ_min_ = −0.18 e Å^−^^3^

### Special details

**Table:**

Geometry. All e.s.d.'s (except the e.s.d. in the dihedral angle between two l.s. planes) are estimated using the full covariance matrix. The cell e.s.d.'s are taken into account individually in the estimation of e.s.d.'s in distances, angles and torsion angles; correlations between e.s.d.'s in cell parameters are only used when they are defined by crystal symmetry. An approximate (isotropic) treatment of cell e.s.d.'s is used for estimating e.s.d.'s involving l.s. planes.
Refinement. Refinement of *F*^2^ against ALL reflections. The weighted *R*-factor *wR* and goodness of fit *S* are based on *F*^2^, conventional *R*-factors *R* are based on *F*, with *F* set to zero for negative *F*^2^. The threshold expression of *F*^2^ > σ(*F*^2^) is used only for calculating *R*-factors(gt) *etc*. and is not relevant to the choice of reflections for refinement. *R*-factors based on *F*^2^ are statistically about twice as large as those based on *F*, and *R*- factors based on ALL data will be even larger.

### Fractional atomic coordinates and isotropic or equivalent isotropic displacement parameters (Å^2^)

**Table:**

	*x*	*y*	*z*	*U*_iso_*/*U*_eq_	
O1	0.59592 (7)	0.23238 (7)	0.23853 (7)	0.01751 (17)	
O2	0.24735 (8)	0.10483 (8)	−0.16272 (8)	0.01954 (17)	
H21	0.2245 (17)	0.1328 (17)	−0.2501 (18)	0.032 (4)*	
N1	0.85431 (9)	0.66853 (8)	−0.05564 (8)	0.01561 (18)	
N2	0.70574 (8)	0.39826 (8)	0.08234 (8)	0.01477 (17)	
N3	0.59208 (9)	0.31588 (9)	0.02612 (9)	0.01608 (18)	
H31	0.5529 (16)	0.3104 (16)	−0.0650 (17)	0.030 (4)*	
N4	0.36471 (8)	0.18113 (8)	−0.09566 (8)	0.01500 (17)	
C1	0.95964 (11)	0.75821 (10)	−0.01987 (11)	0.0176 (2)	
H1	0.9570 (16)	0.8267 (16)	−0.0903 (16)	0.029 (4)*	
C2	1.06781 (10)	0.75321 (10)	0.10549 (10)	0.0168 (2)	
H2	1.1433 (15)	0.8188 (14)	0.1263 (15)	0.023 (3)*	
C3	1.06686 (10)	0.64989 (10)	0.20031 (10)	0.0167 (2)	
H3	1.1409 (16)	0.6414 (16)	0.2873 (16)	0.026 (4)*	
C4	0.95703 (10)	0.55828 (10)	0.16717 (10)	0.01493 (19)	
H4	0.9544 (15)	0.4838 (15)	0.2317 (15)	0.024 (3)*	
C5	0.85205 (10)	0.57099 (9)	0.03849 (9)	0.01315 (18)	
C6	0.73225 (10)	0.47858 (10)	−0.00602 (10)	0.01499 (19)	
H6	0.6756 (15)	0.4825 (15)	−0.1032 (15)	0.026 (3)*	
C7	0.54344 (10)	0.23721 (9)	0.11115 (10)	0.01334 (18)	
C8	0.41629 (10)	0.15545 (9)	0.03573 (10)	0.01366 (19)	
C9	0.36358 (11)	0.05663 (11)	0.11943 (11)	0.0188 (2)	
H9A	0.2794 (19)	0.0181 (19)	0.0675 (19)	0.044 (5)*	
H9B	0.3462 (17)	0.1013 (17)	0.1967 (18)	0.037 (4)*	
H9C	0.4325 (17)	−0.0149 (17)	0.1626 (17)	0.035 (4)*	

### Atomic displacement parameters (Å^2^)

**Table:**

	*U*^11^	*U*^22^	*U*^33^	*U*^12^	*U*^13^	*U*^23^
O1	0.0173 (3)	0.0225 (4)	0.0118 (3)	−0.0006 (3)	0.0036 (3)	0.0006 (2)
O2	0.0176 (4)	0.0249 (4)	0.0133 (3)	−0.0076 (3)	0.0014 (3)	−0.0023 (3)
N1	0.0163 (4)	0.0174 (4)	0.0136 (4)	0.0008 (3)	0.0055 (3)	0.0016 (3)
N2	0.0133 (4)	0.0166 (4)	0.0145 (4)	−0.0022 (3)	0.0046 (3)	−0.0024 (3)
N3	0.0160 (4)	0.0202 (4)	0.0109 (4)	−0.0054 (3)	0.0029 (3)	−0.0011 (3)
N4	0.0128 (4)	0.0168 (4)	0.0146 (4)	−0.0022 (3)	0.0036 (3)	−0.0028 (3)
C1	0.0192 (5)	0.0172 (4)	0.0180 (5)	−0.0002 (3)	0.0084 (4)	0.0029 (3)
C2	0.0155 (4)	0.0163 (4)	0.0198 (5)	−0.0018 (3)	0.0076 (4)	−0.0022 (3)
C3	0.0147 (4)	0.0188 (4)	0.0153 (4)	0.0002 (3)	0.0034 (3)	−0.0017 (3)
C4	0.0161 (4)	0.0153 (4)	0.0132 (4)	0.0001 (3)	0.0046 (3)	0.0007 (3)
C5	0.0139 (4)	0.0138 (4)	0.0128 (4)	0.0006 (3)	0.0058 (3)	−0.0011 (3)
C6	0.0147 (4)	0.0171 (4)	0.0126 (4)	−0.0005 (3)	0.0038 (3)	−0.0014 (3)
C7	0.0133 (4)	0.0139 (4)	0.0133 (4)	0.0011 (3)	0.0050 (3)	−0.0004 (3)
C8	0.0136 (4)	0.0139 (4)	0.0139 (4)	0.0001 (3)	0.0051 (3)	−0.0010 (3)
C9	0.0201 (5)	0.0190 (4)	0.0170 (4)	−0.0037 (4)	0.0058 (4)	0.0024 (3)

### Geometric parameters (Å, °)

**Table:**

O1—C7	1.2249 (13)	C2—H2	0.972 (14)
O2—N4	1.3872 (12)	C3—C4	1.3886 (14)
O2—H21	0.881 (17)	C3—H3	0.959 (15)
N1—C1	1.3427 (13)	C4—C5	1.3967 (15)
N1—C5	1.3505 (12)	C4—H4	0.982 (14)
N2—C6	1.2832 (13)	C5—C6	1.4691 (14)
N2—N3	1.3746 (12)	C6—H6	0.965 (14)
N3—C7	1.3643 (12)	C7—C8	1.5042 (14)
N3—H31	0.879 (16)	C8—C9	1.4962 (13)
N4—C8	1.2848 (14)	C9—H9A	0.930 (19)
C1—C2	1.3854 (16)	C9—H9B	0.962 (17)
C1—H1	0.970 (15)	C9—H9C	0.985 (17)
C2—C3	1.3921 (14)		
			
N4—O2—H21	103.1 (11)	N1—C5—C4	122.25 (9)
C1—N1—C5	117.71 (9)	N1—C5—C6	114.84 (9)
C6—N2—N3	114.32 (9)	C4—C5—C6	122.90 (9)
C7—N3—N2	120.18 (8)	N2—C6—C5	120.45 (9)
C7—N3—H31	119.6 (10)	N2—C6—H6	122.7 (9)
N2—N3—H31	120.2 (10)	C5—C6—H6	116.8 (9)
C8—N4—O2	113.47 (8)	O1—C7—N3	124.18 (9)
N1—C1—C2	123.77 (9)	O1—C7—C8	121.44 (9)
N1—C1—H1	115.0 (9)	N3—C7—C8	114.38 (8)
C2—C1—H1	121.2 (9)	N4—C8—C9	127.58 (9)
C1—C2—C3	118.17 (9)	N4—C8—C7	114.58 (9)
C1—C2—H2	121.1 (9)	C9—C8—C7	117.84 (9)
C3—C2—H2	120.7 (9)	C8—C9—H9A	112.3 (11)
C4—C3—C2	119.06 (10)	C8—C9—H9B	111.8 (10)
C4—C3—H3	120.1 (9)	H9A—C9—H9B	104.7 (15)
C2—C3—H3	120.8 (9)	C8—C9—H9C	111.3 (9)
C3—C4—C5	118.98 (9)	H9A—C9—H9C	111.4 (15)
C3—C4—H4	120.7 (8)	H9B—C9—H9C	104.8 (13)
C5—C4—H4	120.3 (8)		
			
C6—N2—N3—C7	−172.56 (9)	N1—C5—C6—N2	−167.65 (9)
C5—N1—C1—C2	2.31 (14)	C4—C5—C6—N2	13.31 (14)
N1—C1—C2—C3	−0.46 (15)	N2—N3—C7—O1	−1.03 (15)
C1—C2—C3—C4	−1.31 (14)	N2—N3—C7—C8	178.35 (8)
C2—C3—C4—C5	1.19 (14)	O2—N4—C8—C9	−0.54 (14)
C1—N1—C5—C4	−2.42 (14)	O2—N4—C8—C7	−179.80 (7)
C1—N1—C5—C6	178.54 (8)	O1—C7—C8—N4	173.25 (9)
C3—C4—C5—N1	0.71 (14)	N3—C7—C8—N4	−6.14 (12)
C3—C4—C5—C6	179.68 (8)	O1—C7—C8—C9	−6.08 (13)
N3—N2—C6—C5	−178.98 (8)	N3—C7—C8—C9	174.52 (8)

### Hydrogen-bond geometry (Å, °)

**Table:**

*D*—H···*A*	*D*—H	H···*A*	*D*···*A*	*D*—H···*A*
O2—H21···N1^i^	0.881 (17)	1.897 (18)	2.7667 (17)	168.6 (15)
N3—H31···O1^ii^	0.879 (16)	2.217 (16)	2.9656 (15)	142.9 (14)
N3—H31···N4	0.879 (16)	2.240 (16)	2.6059 (15)	104.7 (12)

Symmetry codes: (i) −*x*+1, *y*−1/2, −*z*−1/2; (ii) *x*, −*y*+1/2, *z*−1/2.
